# Cardiovascular effect of nifedipine in morphine dependent rats: hemodynamic, histopathological, and biochemical evidence

**DOI:** 10.3325/cmj.2012.53.343

**Published:** 2012-08

**Authors:** Siyavash Joukar, Mohammad Sheibani, Farzin Joukar

**Affiliations:** 1 Neuroscience Research Center, Kerman University of Medical Sciences, Kerman, Iran; 2 Physiology Research Center, Kerman University of Medical Sciences, Kerman, Iran; 3 Departement of physiology and pharmacology, School of Medicine, Kerman University of Medical Sciences, Kerman, Iran; 4 Kerman Medical Student Research Committee, Kerman University of Medical Sciences, Kerman, Iran; 5 High school of Allame Helli, Education of Kerman province, Kerman, Iran

## Abstract

**Aim:**

To investigate whether administration of nifedipine has considerable therapeutic effect in morphine-dependent rats.

**Methods:**

Sixty animals were randomized into control, morphine, morphine plus nifedipine, and morphine plus dimethyl sulfoxide (DMSO, as nifedipine soluble) groups. Each group consisted of two subgroups, with and without heart injury. The groups were treated with incremental doses of morphine or morphine plus nifedipine daily for 7 days. Myocardial injury was induced by isoproterenol (50 mg/kg i.p.) on the day 7. On the day 8, the heart rate (HR), blood pressure (BP), rate-pressure product (RPP), and the plasma level of cardiac troponin I were measured and the hearts were histopathologically examined.

**Results:**

In morphine-dependent rats, nifedipine administration was associated with a significantly higher decrease in the plasma level of cardiac troponin I than the administration of morphine alone. This finding was also significant in dependent animals that received only DMSO. HR, BP, RPP, and histopathological indices did not show significant changes in the presence of nifedipine.

**Conclusion:**

Administration of nifedipine failed to show a significant therapeutic effect in morphine-dependent rats, especially in the group with myocardial injury.

Acute and chronic morphine administration has cardioprotective effect (1,2). Morphine is also effective in relieving chest pain (3), pulmonary edema, and heart failure after myocardial infarction (4). Morphine dependency is associated with an increase of L-type Ca^2+^ channels' expression, increased calcium entrance to cells, and enhanced basal-free intracellular Ca^2+^ concentration in the central nervous system (5-7).

Calcium and cardiac L-type Ca^2+^ channels have a fundamental role in heart activity (8,9). The calcium channel antagonists/blockers (CCAs/CCBs) are a heterogeneous group of drugs used to treat cardiovascular diseases, including hypertension, angina pectoris, peripheral vascular disorders, and some arrhythmic conditions (10). Three main classes of CCAs include dihydropyridine, phenylalkylamine, and benzothiazepine calcium channel blockers. Dihydropyridine calcium channel blockers such as nifedipine are recommended to reduce systemic vascular resistance and arterial pressure, but their vasodilation and hypotension effects usually lead to reflex tachycardia (11). Phenylalkylamines, eg, verapamil, cause negative inotropy, coronotropy, and dromotropy, reduce myocardial oxygen demand, and reverse coronary vasospasm. Therefore, they are often used to treat angina. They have minimal vasodilatory effects compared with dihydropyridines and cause fewer reflex tachycardias (11). Benzothiazepine calcium channel blockers such as diltiazem show both cardiac depressant and vasodilator actions. They are able to reduce arterial pressure without producing the reflex cardiac stimulation caused by dihydropyridines (11).

The L-type CCBs increase morphine analgesia (12,13) and prevent the development of opioid tolerance and also attenuate the signs of physical dependence (14,15). Despite abundant evidence on the cross interaction between morphine and CCAs effects in central and peripheral nervous system (12-15), the cardiovascular outcome of CCAs administration in morphine dependency has received less attention. However, in some clinical conditions simultaneous long-term administration of calcium channel antagonists and morphine may be inevitable. In a previous study, we demonstrated that sub-chronic co-administration of morphine and verapamil, a phenylalkylamine, had additive cardioprotective effect when compared with each of them alone (16).

Since there is evidence on cardioprotective role of nifedipine on isoproterenol-induced myocardial injury (17), the present study was conducted to elucidate whether nifedipine had cardiovascular effect in morphine-dependent rats, especially those with heart injury.

## Materials and methods

### Chemicals

Morphine sulfate (Temad, Tehran, Iran), sodium thiopental (Biocheme, Kundl, Austria), and isoproterenol (Sigma, Missouri, USA) were dissolved in physiological saline and nifedipine (Sigma) was dissolved in dimethyl sulfoxide (DMSO) plus saline.

### Animal groups

Experiments were performed on 60 male Wistar rats aged 3 months weighing 250-300 g. Animals were randomly divided into five main groups, with no difference in mean weight, and each including two subgroups of rats with or without heart injury. Each subgroup included 7-9 animals. The morphine group was treated with increasing doses of morphine sulfate solution of 10, 10, 12, 15, 15, 20, 20 mg/kg daily i.p. for seven days (16). Morphine dependency was confirmed by withdrawal behaviors following the injection of naloxon HCL 2 mg/kg i.p. to some animals. These behaviors include teeth chattering, chewing, paw tremor, ptosis, writhing, wet-dog shakes, head shakes, diarrhea, ejaculation, erection, and weight loss (18). Morphine + nifedipine (M+NIF) group received nifedipine 10 mg/kg. i.p. daily (19) 30 minutes after morphine injection. M+DMSO group received morphine plus dimethyl sulfoxide (DMSO) but without nifedipine. Control group received equivalent volume of normal saline. Control subgroup that received isoproterenol was called ISO group. Other subgroups that received isoproterenol were added “ISO” to their names (Tables 1 and 2). All experiments followed the guidelines for conducting animal studies (ethics committee permission No 86/123KA – Kerman University of Medical Sciences).

**Table 1:** Blood pressure, heart rate, and pressure rate product in each group of animals

Groups (mean ± standard error of the mean)	Systolic pressure (mmHg)	Diastolic pressure (mmHg)	Mean arterial pressure (mmHg)	Heart rate (beat/min)	Rate-pressure product/1000
**Control (n = 7)**	128 ± 5	94 ± 4	105 ± 4	386 ± 14	41 ± 3
**ISO (n = 7)**	132 ± 6	95 ± 5	107 ± 6	403 ± 8	43 ± 2
**M (n = 9)**	111 ± 7	82 ± 9	92 ± 8	333 ± 13^†^	31 ± 4^†^
**M+ISO (n = 9)**	106 ± 5^‡^	64 ± 5^‡^	78 ± 5^‡^	363 ± 12	29 ± 3^‡^
**M+NIF (n = 7)**	110 ± 8	79 ± 7	90 ± 7	356 ± 16	32 ± 4
**M+NIF+ISO (n = 7)**	109 ± 10^§^	68 ± 9^‡^	82 ± 9^‡^	383 ± 23	31 ± 4^§^
**M+DMSO (n = 7)**	109 ± 9	79 ± 9	89 ± 9	337 ± 22^†^	31 ± 5^†^
**M+DMSO+ISO(n = 7)**	95 ± 7^‡^	59 ± 5^‡║^	71 ± 5^‡^	417 ± 14^¶^**	30 ± 3^‡^

*Abbreviations: M – morphine; NIF – nifedipine; ISO – isoproterenol; DMSO – dimethyl sulfoxide.

†*P* < 0.05 compared with control group.

‡*P* < 0.01 compared with ISO group.

§*P* < 0.05 compared with ISO group.

║*P* < 0.05 compared with M+DMSO group.

¶*P* < 0.01 compared with M+DMSO group.

**P < 0.05 compared with M+ISO group.

**Table 2:** Heart injury histopathological scores in each group of animals*

Groups	No. of animals with myocardial pathology scores^†^						
	n	0	1	2	3	4	Mean
**Control**	7	7	0	0	0	0	0
**ISO**	7	0	0	3	1	3	3^‡^
**M**	9	7	2	0	0	0	0.22
**M+ISO**	9	0	1	5	1	2	2.44^§^
**M+NIF**	7	3	3	0	1	0	0.86
**M+NIF+ISO**	7	0	1	2	2	2	2.71^║^
**M+DMSO**	7	4	1	2	0	0	0.71
**M+DMSO+ISO**	7	0	2	3	1	1	2.14^¶^

*Abbreviations: ISO – isoproterenol; M – morphine; NIF – nifedipine; DMSO –dimethyl sulfoxide.

†0 – nil, 1 – minimum (focal myocytes damage), 2 – mild (small multifocal degeneration with slight degree of inflammatory process), 3 – moderate (extensive myofibrillar degeneration and/or diffuse inflammatory process), 4 – severe (necrosis with diffuse inflammatory process). Although the number of animals with severe lesion (score of 4) was higher in the ISO group, there was no significant difference among animals with heart injury.

‡*P* < 0.01 compared with control.

§*P* < 0.01 compared with M group.

║*P* < 0.05 compared with M+NIF group.

¶*P* < 0.05 compared with M+DMSO group.

### Experimental protocol, sampling, and recording

On the day 7, one hour after having received the last dose of drugs, isoproterenol (50 mg/kg i.p.) was injected to induce cardiac injury (16,20). After 3 hours, a blood sample was taken by retro-orbital puncture from all animals and centrifuged, and serums were stored at -20°C for a maximum of 2 weeks until troponin I, a biochemical marker of myocardial injury, was measured using an enzyme-linked fluorescent immunoassay by VIDAS instrument and the related kit (16).

On the day 8, animals were deeply anesthetized with sodium thiopental (50 mg/kg i.p.) and their trachea was cannulated. During the entire experiment, animals breathed spontaneously. A heparinized saline-filled cannula (15 units/mL) was inserted and fixed into the right carotid artery and subsequently connected to a pressure transducer and physiograph (Beckman R612, Beckman Instr., Schiller Park, IL, USA) for heart rate and arterial blood pressure (BP) recordings. Mean arterial pressure (MAP) was calculated according to “MAP=Pd+ (Ps-Pd)/3 formula,” where Pd stands for diastolic and Ps for systolic arterial pressure. Rate-pressure product (RPP), an index of myocardial oxygen demand, was estimated by the product of the heart rate and mean arterial pressure [(MAP*heart rate)/1000] (21,22).

### Histopathological study

After heart rate and blood pressure recordings had been made, the animals were killed under deep anesthesia and their hearts were removed, fixed in 10% buffered formalin, and embedded in paraffin after tissue processing. Five micron-thick sections were prepared, stained with hematoxylin and eosin, and examined microscopically by two pathologists blinded to animal grouping. The lesions were graded as 0) nil; 1) minimum (focal myocytes damage); 2) mild (small multifocal degeneration with slight degree of inflammatory process); 3) moderate (extensive myofibrillar degeneration and/or diffuse inflammatory process); and 4) severe (necrosis with diffuse inflammatory process) (23).

### Statistical analysis

The values are expressed as mean ± standard error of the mean. Data analysis was performed by SPSS, version 14 (SPSS Inc., Chicago, IL, USA). One-way ANOVA followed by post hoc Tukey test was used to compare the quantitative data. Comparisons of histopathological findings were carried out using the non parametric Kruskal-Wallis and pairwise differences by the Mann-Whitney U-test (16). *P* value <0.05 was considered as statistically significant.

## Results

### Hemodynamic findings

There was a non significant reduction in arterial blood pressure in the morphine subgroup without injury compared to control group, regardless of nifedipine or DMSO treatment (Table 1). However, in the heart injury subgroup, morphine, M+NIF, and M+DMSO groups showed a significant reduction in systolic, diastolic, and MAP. Morphine with and without DMSO decreased the heart rate compared to control group (*P* < 0.05), but DMSO increased it in animals with heart injury.

In addition, all morphine-dependent animals with heart injury, M+ISO, M+DMSO+ISO, and M+NIF+ISO showed a significant reduction in RPP when compared with ISO group (Table 1). On the other hand, there was no significant difference in RPP value between morphine group and nifedipine + morphine group, both in animals with and without heart injury.

### Plasma cardiac troponin I levels

**Figure 1:**
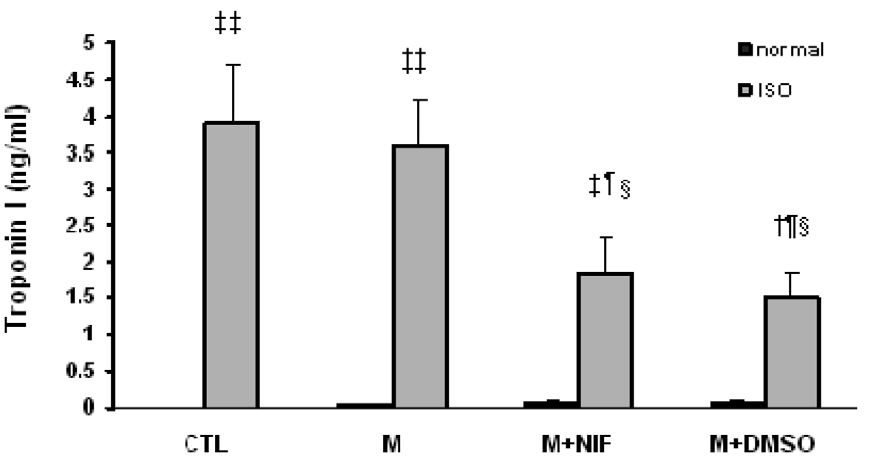
Plasma cardiac troponin I levels in groups of animals. The results are presented are mean ± standard error of the mean. n = 6-7, normal: subgroup without isoproterenol-induced cardiac injury; ISO – subgroup with isoproterenol induced cardiac injury; CTL – control; M – morphine; NIF – nifedipine; DMSO – dimethyl sulfoxide. ^†^*P* < 0.05, ^‡^*P* < 0.01, ^‡‡^*P* < 0.001 compared with relative normal group. ^¶^*P* < 0.01 compared with the corresponding CTL group. ^§^*P* < 0.01 compared with morphine group. Normal – closed bars; ISO – gray bars.

Cardiac injury was associated with a significant increase in plasma cardiac troponin I levels in all animal groups when compared with their corresponding control subgroups. This increase was lower in M+DMSO (*P* < 0.05) and M+NIF subgroups (*P* < 0.01 vs their corresponding control subgroups) and was higher for M and control subgroups (*P* < 0.001 vs their corresponding control subgroups) (Figure 1). Comparisons among injury subgroups showed a significant decline in troponin I levels in dependent animals in the presence of DMSO or nifedipine when compared with control or morphine groups (*P* < 0.01). However, animals with morphine dependency alone did not show a significant difference compared to control group (Figure 1).

### Histopathological findings

**Figure 2:**
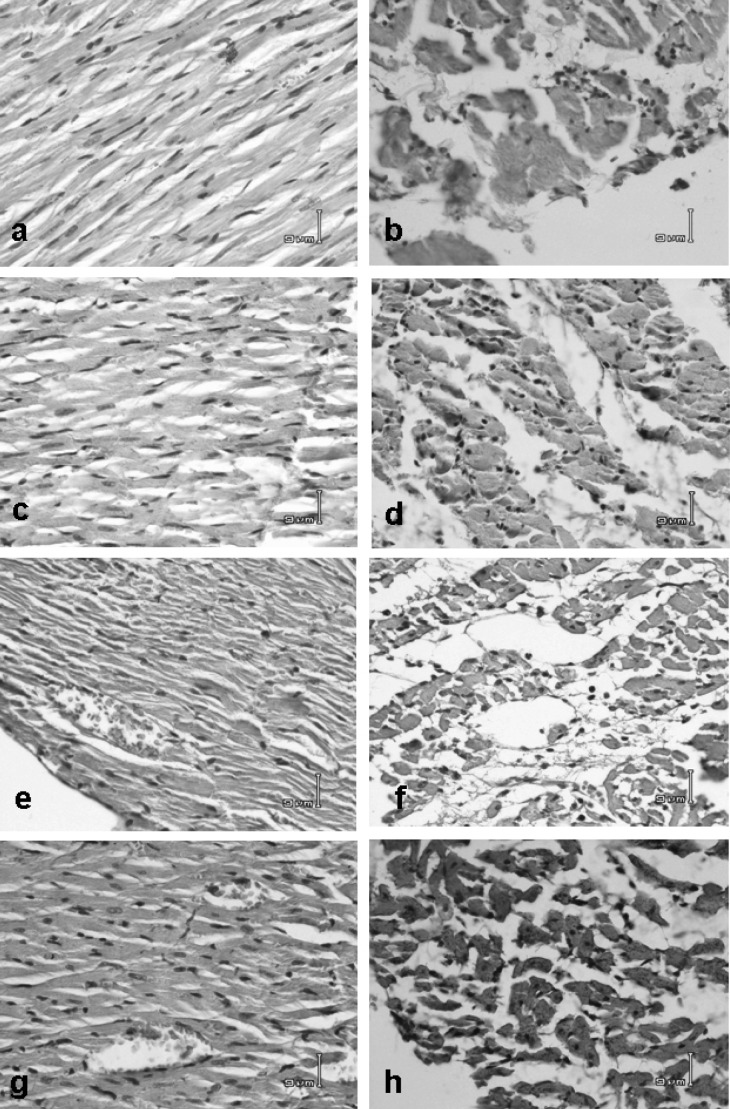
Hematoxylin and eosin stained sections of heart tissue in different animal groups. (**A**) Control group heart section with normal appearance of cardiac myofibers. (**B**) Control group that received isoproterenol (ISO) showing severe myocardial degeneration. (**C**) Normal architecture of myocytes in morphine group. (**D**) Degeneration, edema, and moderate degree of fibroblastic proliferation and inflammatory process in morphine subgroup that received isoproterenol. (**E**) Heart sections from morphine + nifedipine group (M+NIF) showing prominence coronary vasodilatation. (**F**) A section from M+NIF after isoproterenol-induced heart injury. Degeneration, edema, inflammatory process, and fibroblastic proliferation are obvious. (**G**) and (**H**) are sections from morphine+DMSO (M+DMSO) subgroups with/without myocardial injury, respectively.

Control group showed normal appearance of myocardial tissue. Isoproterenol injection was associated with varying degrees of muscle heart damage in different groups (*P* < 0.01 for ISO and M+ISO vs control and M groups, respectively, and *P* < 0.05 for other groups compared to relative control). The highest level of damage was observed in the control subgroup with cardiac injury. In this subgroup, 50% of animals showed cellular necrosis with diffuse inflammatory process (Table 1). On the other hand, pre-treatment with morphine, alone and concomitant with nifedipine or DMSO, non significantly attenuated the severity of myocardial damage (Table 2 and Figure 2). In addition, coronary dilatation and tissue congestion were dominant phenomena in animals that were treated with morphine with or without nifedipine, especially in heart injury groups (Figure 2).

## Discussion

This study found that nifedipine had a mild cardioprotective role in rats with heart injury, which was enhanced by DMSO, and did not have a significant effect on hemodynamic, myocardial oxygen consumption and biochemical and histopathological indicators in morphine-dependent rats with or without heart injury.

The cardioprotective effect of morphine, confirmed by lower levels of myocardial damage, was also found in previous studies (1,2). However, morphine had no effect on serum cardiac troponin I level as an important biomarker of cardiac damage.

Morphine fails to reduce LDH release following ischemia/reperfusion but improves heart contractility (2). In addition, in two previous studies we found that opium decreased isoproterenol-induced heart lesions but was unable to reduce serum levels of cardiac troponin I (24,25). Moreover, it was reported that morphine therapy in acute decompensated heart failure significantly increased serum cardiac troponin I levels (26).

The mismatch between troponin I level and the extent of heart damage in morphine-dependent animals was observed in previous studies (16,24-26), as well as in this study. However, to the best of our knowledge, no study explained this inconsistency and further studies are needed. The cardioprotective effect of morphine may be attributed to peripheral vasodilatation and negative chronotropy secondary to vagal tone amplification and sympathetic tone diminution by morphine (27), which in turn reduces the cardiac workload and oxygen demand. This study showed that isoproterenol increased the heart rate and RPP to some extent. However, chronic morphine use decreased the heart rate and RPP and also induced coronary vasodilatation, as judged by coronary dilatation in histological examination.

The question remains as to why the combination of morphine and nifedipine did not show excess cardioprotective effect. Nifedipine consumption is associated with reflex tachycardia, which occurs due to the vasodilatation and hypotension effects of this drug (11) and hence is not usually prescribed to treat angina. Our finding is consistent with previous findings on reflex tachycardia effect of nifedipine. There was an increased trend in heart rate and RPP in M+NIF group with/without heart injury when compared with the corresponding morphine group. However, these differences were not significant and the collective effect of these two changes may be attenuated by the positive effect of morphine as appeared in histopathological findings. The other possibility is the interaction of morphine and nifedipine on heart L-type Ca^2+^ channels. In the central nervous system, chronic use of morphine enhances the L-type Ca^2+^ channels' expression, augments the calcium entry in to cell, and increases basal free intracellular Ca^2+^ concentration (5-7). Other effects of morphine are prolongation of action potential by augmentation of L-type calcium current (28) and increase in heart myofilament sensitivity to calcium (29). On the other hand, chronic administration of nifedipine up-regulates L-type calcium channels receptors of the heart (30,31). This recently discovered effect of nifedipine, along with reflex tachycardia, can increase the concentration of free intracellular calcium that is triggered by morphine and hence modulate the cardioprotective effect of morphine.

It seems that the reduction of troponin I level in M+NIF group does not result from nifedipine effect, but is arising from solvent (DMSO) effect. Because of this, the consumption of DMSO was associated with a decrease in both troponin I level and the severity of heart damage. In addition, the histopathological index is a more reliable indicator of the extent and severity of heart damage than other indices.

This finding is also consistent with studies that have shown neuroprotective (32,33) and cardioprotective (33) role of DMSO. It is reported that DMSO has an effect on blocking Na^+^ and Ca^2+^ entry into the cells (34,35). Since a high dose of isoproterenol causes cytosolic calcium overload that mediated through the calcium channels, DMSO administration may prevent this inward cellular ion flux and hence attenuate heart damage.

In conclusion, our study suggested that in the presence of morphine dependency, nifedipine administration had no considerable cardiovascular effect. This finding may give a new perspective on the application of dihydropyridine calcium channel blockers in narcotic dependent patients.

## References

[1] Gross ER, Hsu AK, Gross GJ (2004). Opioid-induced cardioprotection occurs via glycogen synthase kinase beta inhibition during reperfusion in intact rat hearts.. Circ Res.

[2] Peart JN, Gross GJ (2004). Chronic exposure to morphine produces a marked cardioprotective phenotype in aged mouse hearts.. Exp Gerontol.

[3] Shannon AW, Harrigan RA (2001). General pharmacologic treatment of acute myocardial infarction.. Emerg Med Clin North Am.

[4] Peacock WF, Hollander JE, Diercks DB, Lopatin M, Fonarow G, Emerman CL (2008). Morphine and outcomes in acute decompensated heart failure: an ADHERE analysis.. Emerg Med J.

[5] Katsura M, Ohkuma S (2004). PharmacologicabBasis for management of drug dependence.. Ann N Y Acad Sci.

[6] Diaz A, Ruiz F, Florez J, Pazos A, Hurle MA (1995). Regulation of dihydropyridine-sensitive Ca2+ channels during opioid tolerance and supersensitivity in rats.. J Pharmacol Exp Ther.

[7] Diaz A, Florez J, Pazos A, Hurle MA (2000). Opioid tolerance and supersensitivity induce regional changes in the autoradiographic density of dihydropyridin-esensitive calcium channels in the rat central nervous system.. Pain.

[8] Richard S, Perrier E, Fauconnier J, Perrier R, Pereira L, Gomez AM (2006). 'Ca2+ -induced Ca2+ entry' or how the L-type Ca2+ channel remodels its own signalling pathway in cardiac cells.. Prog Biophys Mol Biol.

[9] Bers DM, Despa S (2006). Cardiac myocytes Ca2+ and Na+ regulation in normal and failing hearts.. J Pharmacol Sci.

[10] Triggle DJ (2007). Calcium channel antagonists: Clinical uses – past, present and future.. Biochem Pharmacol.

[11] Brunton L, Chabner B, Knollman B. Goodman & Gilmans the pharmacologicalbasis of theraputics, 12th edition. New York (NY): McGraw – Hill; 2011.

[12] Shimizu N, Kishioka S, Maeda T, Fukazawa Y, Dake Y, Yamamoto C (2004). Involvement of peripheral mechanism in the verapamil-induced potentiation of morphine analgesia in mice.. J Pharmacol Sci.

[13] Dogrul A, Yesilyurt O, Isimer A, Guzeldemir ME (2001). L-type and T-type calcium channel blockade potentiate the analgesic effects of morphine and selective mu opioid agonist, but not to selective delta and kappa agonist at the level of the spinal cord in mice.. Pain.

[14] Smith FL, Dombrowski SD, Deway LW (1999). Involvement of intracellular calcium in morphine tolerance in mice.. Pharmacol Biochem Behav.

[15] Michaluk J, Karokewicz B, Antkiewicz-Michaluk L, Vetulani J (1998). Effect of various Ca2+ channel antagonists on morphine analgesia, tolerance and dependence, and on blood pressure in the rat.. Eur J Pharmacol.

[16] Joukar S, Najafipour H, Dabiri S, Sheibani V, Esmaeili-Mahani S, Ghotbi P (2011). The effect of chronic co-administration of morphine and verapamil on isoproterenol-induced heart injury.. Cardiovasc Hematol Agents Med Chem.

[17] Chen NH, Rao MR (1990). Protecting effects of m-nifedipine on isoproterenol-induced myocardial injury in rats.. Zhongguo Yao Li Xue Bao..

[18] Joukar S, Atapour N, Kalantaripour T, Bashiri H, Shahidi A (2011). Differential modulatory actions of GABAA agonists on susceptibility to GABAA antagonists-induced seizures in morphine dependent rats: Possible mechanisms in seizure propensity.. Pharmacol Biochem Behav.

[19] Morgan PE, Aiello EA, Chiappe de Cingolani GE, Mattiazzi AR, Cingolani HE (1999). Chronic administration of nifedipine induces up regulation of functional calcium channels in rat myocardium.. J Mol Cell Cardiol.

[20] York M, Scudamore C, Brady S, Chen C, Wilson S, Curtis M (2007). Characterization of troponin responses in isoproterenol-induced cardiac injury in the hanover wistar rat.. Toxicol Pathol.

[21] Gobel FL, Norstrom LA, Nelson RR, Jorgensen CR, Wang Y (1978). The rate-pressure product as an index of myocardial oxygen consumption during exercise in patients with angina pectoris.. Circulation.

[22] Polakowski JS, King AJ, Campbell TJ, Nelson RA, Preusser LC, Kempf-Grote AJ (2009). Cardiovascular effects of torcetrapib in conscious and pentobarbital-anesthetized dogs.. J Cardiovasc Pharmacol.

[23] Joukar S, Najafipour H, Khaksari M, Sepehri G, Shahrokhi N, Dabiri S (2010). The effect of saffron consumption on biochemical and histopathological heart indices of rats with myocardial infarction.. Cardiovasc Toxicol.

[24] Najafipour H, Joukar S, Malekpour-Afshar R, Mirzaeipour F, Nasri HR (2010). Passive opium smoking does not have beneficial effect on plasma lipids and cardiovascular indices in hypercholesterolemic rabbits with ischemic and non-ischemic hearts.. J Ethnopharmacol.

[25] Joukar S, Najafipour H, Malekpour-Afshar R, Mirzaeipour F, Nasri HR (2010). The effect of passive opium smoking on cardiovascular indices of rabbits with normal and ischemic hearts.. Open Cardiovasc Med J..

[26] Peacock WF, Hollander JE, Diercks DB, Lopatin M, Fonarow G, Emerman CL (2008). Morphine and outcomes in acute decompensated heart failure: an ADHERE analysis.. Emerg Med J.

[27] Zipes DP, Libby P, Bonow OR, Braunwald E. Braunwald's heart disease: a text book of cardiovascular medicine, 8th ed., Vol. 2, Philadelphia (PA): Saunders; 2008.

[28] Xiao GS, Zhou JJ, Wang GY, Cao CM, Li GR, Wong TM (2005). In vitro electrophysiologic effects of morphine in rabbit ventricular myocytes.. Anesthesiology.

[29] Nakae Y, Fujita S, Namiki A (2001). Morphine enhances myofilament ca2+ sensitivity in intact guinea pig beating hearts.. Anesth Analg.

[30] Chiappe De Cingolani GE, Mosca SM, Vila Petroff M, Cingolani HE (1994). Chronic administration of nifedipine induces upregulation of dihydropyridine receptors in rabbit heart.. Am J Physiol.

[31] Morgan PE, Aiello EA, Chiappe de Cingolani GE, Mattiazzi AR, Cingolani HE (1999). Chronic administration of nifedipine induces up regulation of functional calcium channels in rat myocardium.. J Mol Cell Cardiol.

[32] Bardutzky J, Meng X, Bouley J, Duong TQ, Ratan R, Fisher M (2005). Effects of intravenous dimethyl sulfoxide on ischemia evolution in a rat permanent occlusion model.. J Cereb Blood Flow Metab.

[33] Jacob SW, de la Torre JC (2009). Pharmacology of dimethyl sulfoxide in cardiac and CNS damage.. Pharmacol Rep.

[34] Camici GG, Steffel J, Akhmedov A, Schafer N, Baldinger J, Schulz U (2006). Dimethyl sulfoxide inhibits tissue factor expression, thrombus formation, and vascular smooth muscle cell activation: a potential treatment strategy for drug-eluting stents.. Circulation.

[35] Hulsmann S, Greiner C, Kohling R (1999). Dimethyl sulfoxide increases latency of anoxic terminal negativity in hippocampal slices of guinea pig in vitro.. Neurosci Lett.

